# A Novel Azimuth Super-Resolution Method by Synthesizing Azimuth Bandwidth of Multiple Tracks of Airborne Stripmap SAR Data

**DOI:** 10.3390/s16060869

**Published:** 2016-06-13

**Authors:** Yan Wang, Jingwen Li, Bing Sun, Jian Yang

**Affiliations:** 1 Department of Electronic Engineering, Tsinghua University, Beijing 100084, China; yan_wang@mail.tsinghua.edu.cn; 2 School of Electronic and Information Engineering, Beihang University, Beijing 100191, China; lijingwen@buaa.edu.cn (J.L.); bingsun@buaa.edu.cn (B.S.)

**Keywords:** airborne stripmap SAR, azimuth super-resolution, bandwidth synthesization, multiple tracks

## Abstract

Azimuth resolution of airborne stripmap synthetic aperture radar (SAR) is restricted by the azimuth antenna size. Conventionally, a higher azimuth resolution should be achieved by employing alternate modes that steer the beam in azimuth to enlarge the synthetic antenna aperture. However, if a data set of a certain region, consisting of multiple tracks of airborne stripmap SAR data, is available, the azimuth resolution of specific small region of interest (ROI) can be conveniently improved by a novel azimuth super-resolution method as introduced by this paper. The proposed azimuth super-resolution method synthesize the azimuth bandwidth of the data selected from multiple discontinuous tracks and contributes to a magnifier-like function with which the ROI can be further zoomed in with a higher azimuth resolution than that of the original stripmap images. Detailed derivation of the azimuth super-resolution method, including the steps of two-dimensional dechirping, residual video phase (RVP) removal, data stitching and data correction, is provided. The restrictions of the proposed method are also discussed. Lastly, the presented approach is evaluated via both the single- and multi-target computer simulations.

## 1. Introduction

Synthetic Aperture Radar (SAR) is a multi-platform multi-mode microwave imaging tool that equalizes its trajectory to a wide aperture antenna to generate high resolution images [1,2,3]. Airborne SAR can image a region of interest (ROI) with flexible tracks, little response time and low transmitting power and hence has been playing important roles in wide applications, e.g., disaster monitoring, moving target direction and navigation assistance [4,5,6,7,8,9]. Stripmap mode is the most basic SAR operation mode and is often used to image long strips along the aircraft track. However, as the stripmap mode employs no azimuth steering, the azimuth resolution of which is at most equal to half of the azimuth antenna size [2]. If a higher azimuth resolution is required, other modes, e.g., the spotlight mode, with a wider synthetic antenna aperture realized by the continuous beam steering, should be used instead.

In practical applications, it is often found that richer details of some areas within focused stripmap images, like vehicles or artificial architectures surrounded by forests, are needed with a higher azimuth resolution. The typical strategy tends to send another sortie of aircraft to collect data one more time with a higher-azimuth-resolution mode. Such strategy is time-consuming and cost-ineffective but reasonable if only a single track of stripmap SAR data is available. However, if multiple tracks of the stripmap data have already been achieved for interferometric SAR (InSAR) applications, an innovative azimuth super-resolution method, as will be discussed in this paper, is able to improve the azimuth resolution of any given small ROI. The function of this azimuth super-resolution method is similar to the way a magnifier works: any small patch within the original stripmap SAR image can be further zoomed-in to achieve a higher azimuth resolution than that of the original stripmap mode. Thus, the endeavor of arranging another flight for the data collection can be avoided.

Current super-resolution researches focus on two aspects: the first is a range super-resolution technique that synthesizes the sub-bandwidths of transmitted signals to form an ultra-wide range bandwidth [10,11,12,13,14,15]; the second is an elevation super-resolution technique that employs the theory of compressive sensing to generate tomographic three-dimension SAR images [16,17,18,19]. The motivation of the proposed azimuth super-resolution method differs a lot from that of the above-mentioned super-resolution research. For almost all existing super-resolution methods, the improvement of the resolution performance relies on updates of the original hardware and platforms, hence resulting in a higher cost. The proposed azimuth resolution method, however, improves the azimuth resolution by employing multiple tracks of data instead of updated hardware or platforms, and hence is more cost-effective.

The core idea of the proposed azimuth super-resolution method is to expand the azimuth bandwidth of the signal in the wavenumber domain via resorting and stitching the data of multiple tracks in order. The procedure of the azimuth super-resolution method consists of five steps. Firstly, a two dimensional dechirping is employed to implement a unique mapping between the time and the frequency domain. Second, a range frequency domain filtering is induced to remove the quadratic residual video phase (RVP). Third, the data of selected multiple tracks are stitched together with respect to their geometrical positions. Fourth, two cascade interpolations along the range and the azimuth directions are employed to convert the dechirped data from a polar format to a rectangular format. Finally, two cascade one-dimensional (1D) Fast Fourier Transforms (FFT) in range and in azimuth, respectively, are used to complete the data focusing. The restrictions of the proposed azimuth super-resolution method are discussed subsequently.

This paper is organized as follows. In Section 2, the data acquisition geometry and the signal model are introduced. In Section 3, the azimuth super-resolution method is presented and the mathematical formulae are derived. Section 4 discusses the restrictions of the presented approach. The effectiveness of the presented azimuth super-resolution method is evaluated via the computer simulation experiments in Section 5. Section 6 summarizes this paper and suggests future work.

## 2. Geometry and Signal Model

**Figure 1:**
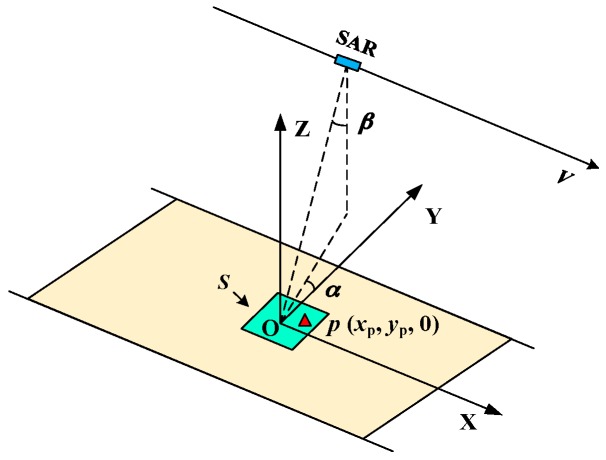
Geometry of the stripmap data acquisition.

The geometry of the stripmap data acquisition is shown in Figure 1. *S* is assumed to be the area requiring a higher azimuth resolution. A three-dimensional (3D) Cartesian coordinate is built with an origin *O* locating at the center of *S*, an *x*-axis along the track of the sensor, a *z*-axis vertical to the ground surface and a *y*-axis formed by the right hand rule. The look angle, denoted as *β*, is formed by the nadir direction and the origin-to-sensor direction. The gazing angle, denoted as *α*, is formed by the ground projection of the slant range to the origin and the *y*-axis. *p* (*x*_p_, *y*_p_, 0) denotes an arbitrary target within the area *S*.

By assuming a linear frequency-modulated (LFM) signal is transmitted by pulse as [1,2,20]

(1) $$s_{t}\left(\tau \right)=\text{rect}\left[\frac{\tau }{T_{p}}\right]\exp\left\{j2\pi f_{c}\tau +j\pi \gamma \tau^{2}\right\}$$

where *f*_c_ is the carrier frequency, *T*_p_ is the pulse length and *γ* is the chirp rate of the LFM signal, *τ* denotes the fast range time and rect[·] denotes the rectangular function. As the amplitude of the signal has no effect on focusing, it is neglected in Equation (1) and in subsequent derivations. By assuming that *R* is the slant range from the sensor to target *p*, the backscattered signal mixed back to the baseband can be digitally recorded as [1,2,20]

(2) $$s_{r}\left(m,i\right)=\text{rect}\left[\frac{i}{N_{r}}\right]\exp\left\{j\pi \gamma {\left(\frac{i}{F_{s}}-\frac{2R\left(m,p\right)}{c}\right)}^{2}-j\frac{4\pi f_{c}R\left(m,p\right)}{c}\right\},\left(m=-\frac{N_{a}}{2},...,\frac{N_{a}}{2},i=-\frac{N_{r}}{2},...,\frac{N_{r}}{2}\right)$$

where *F*_s_ denotes the sampling frequency, *N*_r_ and *N*_a_ denote the range and the azimuth sampling points, respectively.

## 3. Derivation of the Azimuth Super-Resolution Method

The azimuth super-resolution method proposed here is realized by directly manipulating the raw data of discontinuous tracks instead of using focused stripmap images. Hence, the implementation of the presented azimuth super-resolution should be viewed as raw data imaging, aiming at generating SAR images of small areas using the data with discontinuous slant ranges from different tracks.

### 3.1. Two Dimensional Dechirping

From Equation (2), it is known that the discontinuous slant ranges will hamper the azimuth LFM characteristics of the signal and hence will disable almost all Doppler-based imaging algorithms. Thus, the implementation of the azimuth super-resolution method should not rely on the azimuth LFM feature. Therefore, the first step is to employ a two dimensional dechirping processing to remove the two dimensional LFM feature of individual tracks. The LFM feature removal can be realized by multiplying the conjugate replica of the echo backscattered from the scene center as

(3) $$s_{\text{ref}}\left(m,i\right)=\exp\left\{-j\pi \gamma {\left(\frac{i}{F_{s}}-\frac{2R_{c}\left(m\right)}{c}\right)}^{2}+j\frac{4\pi f_{c}R_{c}\left(m\right)}{c}\right\}$$

where *R*_c_ denotes the slant range from the SAR sensor to the scene center. After the two dimensional dechirping processing, the signal yields

(4) $$\begin{array}{l} s_{\text{if}}\left(m,i\right)=\text{rect}\left[\frac{i}{N_{r}}\right]\exp\left\{j\frac{4\pi \gamma }{c^{2}}{\left(R\left(m,p\right)-R_{c}\left(m\right)\right)}^{2}\right\}\\ \;\;\;\;\cdot \exp\left\{-j\frac{4\pi }{c}\left(f_{c}+\gamma \left(\frac{i}{F_{s}}-\frac{2R_{c}\left(m\right)}{c}\right)\right)\left(R\left(m,p\right)-R_{c}\left(m\right)\right)\right\} \end{array}$$

**Figure 2:**
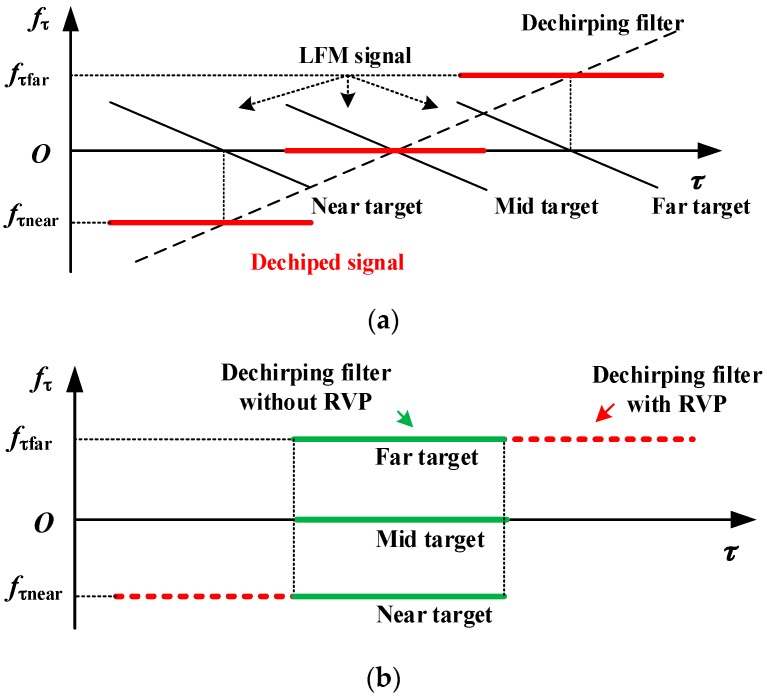
Effects of the (**a**) two dimensional dechirping; and (**b**) residual video phase removal to the signal distributions in the time-frequency domain.

Comparing with Equation (2), it can be found that Equation (4) has been converted from an LFM signal to a monochrome signal, the frequency of which is uniquely determined by the target position, as the coordinate of *p* for the derivation above. In this way, the target positions are uniquely mapped to individual frequencies as shown in the time-frequency domain (TFD) in Figure 2a, where *f*_τ_ denotes the range frequency. Three targets, located at the near, middle and far ranges, are illustrated. As a result, the wavenumber domain signal processing can be equivalently realized in the time domain.

### 3.2. RVP Removal

The first quadratic term in Equation (4) is called RVP, which leads to a staggered distribution of the dechirped signal in the TFD as shown in Figure 2a [1]. If not compensated, the RVP will deteriorate the quality of the focused image in azimuth. Due to Figure 2a, it is known that a range FFT can directly focus the two dimensional dechirped monochromatic signal in range, resulting in a range frequency *f*_τp_ where the peak value of the focused sinc function locates as [1]

(5) $$f_{\tau p}\left(m,p\right)=\frac{2\gamma }{c}\left(R\left(m,p\right)-R_{c}\left(m\right)\right)$$

By substituting Equations (5) into (4), the expression of the RVP in the range frequency domain can be achieved, the conjugate expression of which can be used as a RVP removal filter as

(6) $$f_{\text{RVP}}\left(i\right)=\exp\left\{-j\pi \frac{i^{2}}{\gamma F_{s}^{2}}\right\}$$

After being filtered by *f*_RVP_, the signal is converted back to the range time domain as

(7) $$s_{\text{if}1}\left(m,i\right)=\text{rect}\left[\frac{i}{N_{r}}\right]\exp\left\{-j\frac{4\pi }{c}\left(f_{c}+\gamma \left(\frac{i}{F_{s}}-\frac{2R_{c}\left(m\right)}{c}\right)\right)\left(R\left(m,p\right)-R_{c}\left(m\right)\right)\right\}$$

**Figure 3:**
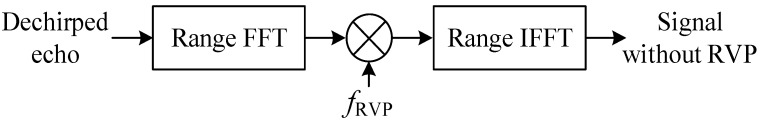
Flowchart of the RVP removal manipulation.

The distribution of the dechirped signal without RVP, expressed by Equation (7), in the TFD is displayed in Figure 2b, from which it can be seen that the signals with different slant ranges are aligned along the range (time) direction. The flowchart of the RVP removal manipulation is shown in Figure 3.

Given that the target *p* has a zero-altitude, the phase of Equation (7) can be rewritten under a planar wavefront approximation as [20,21,22]

(8) $$s_{\text{if}1}\left(m,i\right)=\exp\left\{-j\left[K_{x}\left(m,i\right)x_{p}+K_{y}\left(m,i\right)y_{p}\right]\right\}$$

where

(9) $$K_{x}\left(m,i\right)=\frac{4\pi }{c}\left(f_{c}+\frac{\gamma }{F_{s}}i\right)\sin\beta \left(m\right)\sin\alpha \left(m\right)$$

(10) $$K_{y}\left(m,i\right)=\frac{4\pi }{c}\left(f_{c}+\frac{\gamma }{F_{s}}i\right)\sin\beta \left(m\right)\cos\alpha \left(m\right)$$

where *K*_x_ and *K*_y_ denote the azimuth wavenumber and the range wavenumber, respectively. Equation (8) is the foundation for the subsequent data stitching and correcting manipulation.

### 3.3. Data Stitching and Requirement

**Figure 4:**
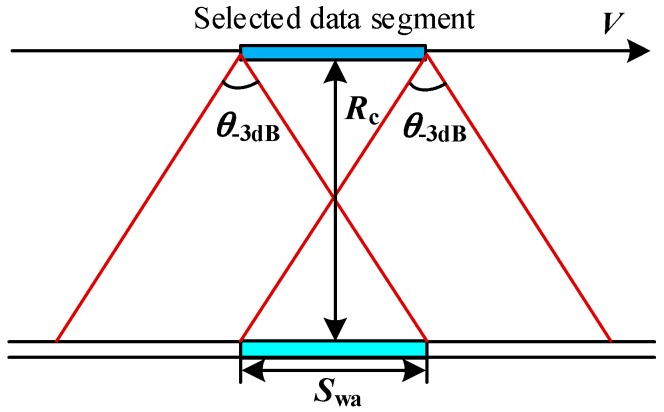
Track segment selection for the data stitching.

Two rules should be satisfied to ensure the effectiveness of the proposed azimuth super-resolution method. Firstly, the entire ROI should be continuously illuminated within the selected track segment to ensure that the wavenumber spectrum of each target within the ROI is complete. This requirement is equivalent to generating a spotlight-mode echo using a stripmap-mode antenna. Thus, the maximum effective azimuth swath of the target region, denoted as *S*_wa_, would be limited by the −3dB ground wave footprint in azimuth as

(11) $$S_{\text{wa}}<R_{c}\theta_{-3\text{dB}}$$

where *θ*_−3dB_ denotes the azimuth −3dB antenna width. This is the reason why the swath upon which the azimuth super-resolution method can be successfully applied should be small. Figure 4 illustrates this limitation, which implies that the azimuth resolution of each selected data segment would be worse than that of the original stripmap data. However, by resorting and stitching the data segments of multiple tracks together based on an increasing gazing angle criterion, a higher azimuth resolution than that of the stripmap mode can be achieved by expanding the signal wavenumber spectrum in azimuth. In practical application, such data extraction should be a trade-off between the effective azimuth swath and the azimuth resolution: the shorter the effective azimuth swath, the higher the azimuth resolution that could be achieved. For the proposed method, such “azimuth resolution scarification” should be regarded as an inevitable compromise for the azimuth wavenumber bandwidth expansion.

**Figure 5:**
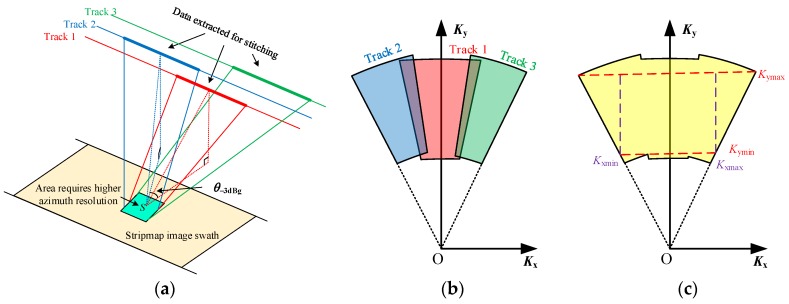
Illustrations of (**a**) the data selection; the wavenumber spectrums of (**b**); the selected data segments; and (**c**) the stitched data.

Secondly, in the case of a common parallel-track assumption, the sensors of different tracks are required to operate with slightly different squint angles to form staggered spatial sampling positions as shown in Figure 5a. Based on the unique mapping between the time and wavenumber domain as discussed above, a staggered wavenumber spectrum can be correspondingly generated as shown in Figure 5b. The non-overlapping areas of the staggered wavenumber distribution denote the potential of the extent to which the azimuth spectrum can be expanded. As the spatial varieties of *K*_x_ and *K*_y_ are only determined by the gazing angle *α* and the look angle *β* as shown in Equations (9) and (10), a reasonable data stitching method can be employed to resort the data of different tracks with regard to the gazing angle *α*. Thus, a stitched and combined wavenumber spectrum can be achieved as shown in Figure 5c, of which the azimuth wavenumber spectrum bandwidth is wider than that of any individual track. The rule of arranging the squint angles of different tracks will be further discussed in Section 4.

It has to be stated that, despite inconsistent squint angles of different tracks, the multi-track data can still be employed for InSAR applications as mentioned in Section 1. However, unlike the processing of the traditional interferometric SAR data with consistent squint angles, the processing of the data acquired by antiparallel tracks, *i.e.*, with inconsistent squint angles, would need an additional coregistration step [23]. As long as the beam direction is properly set for individually tracks, their data can be effectively stitched to generate higher-azimuth-resolution SAR images. Thus, the motivation of the proposed azimuth super-resolution method in cutting down the data acquirement cost can still be satisfied.

### 3.4.Two Dimensional Data Correction

The wavenumber spectrum of the stitched data in Figure 5c shows that both *K*_x_ and *K*_y_ are spatial variants and should be corrected for the decoupling between the range and the azimuth directions. The correction method is inspired by a polar format algorithm (PFA) but is further developed to agree with the features of the stitched data.

A range interpolation is firstly conducted to remove the spatial variety of the range wavenumber. Different from the conventional range interpolation processing of the PFA that employs the central range wavenumber as the range referential interpolation array [1], the range interpolation designed here extracts a uniform inscribed region within the stitched spectrum to generate the range referential interpolation array. By assuming that *K*_ymax_ is the minimum value of the upper boundary of the stitched range spectrum and *K*_ymin_ is the maximum value of the lower boundary of the stitched range spectrum as shown in Figure 5c, the range referential interpolation array *K*_yref_ yields

(12) $$K_{\text{yref}}\left(i_{1}\right)=K_{\text{ymin}}+\frac{K_{\text{ymax}}-K_{\text{ymin}}}{N_{r}}i_{1},\;\left(i_{1}=1,2,...,N_{r}\right)$$

**Figure 6:**
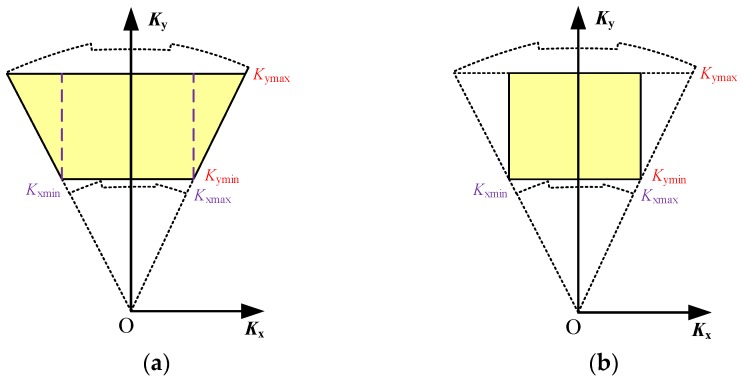
Wavenumber spectrum of the stitched data (**a**) after the range interpolation; and (**b**) after the azimuth interpolation.

By keeping the range sample number unchanged as *N*_r_, *K*_yref_ is actually resampled with a higher sampling rate than *F*_s_ in Equation (8) because the range wavenumber bandwidth is slightly compressed due to the selected inscribed wavenumber spectrum. Therefore, no signal aliasing in range will occur. The range interpolation converts the data from the polar format to the keystone format (in yellow) as shown in Figure 6a, resulting in a signal format as

(13) $$s_{\text{if}2}\left(m_{1},i_{1}\right)=\exp\left\{-j\left[K_{x1}\left(m_{1},i_{1}\right)x_{p}+K_{\text{yref}}\left(i_{1}\right)y_{p}\right]\right\}$$

where

(14) $$K_{x1}\left(m_{1},i_{1}\right)=\tan\alpha \left(m_{1}\right)K_{\text{yref}}\left(i_{1}\right),\;\left(m_{1}=1,2,...,N_{a1}\right)$$

where *N*_a1_ is the new azimuth sample number of the stitched data.

For the next step, the dependence of the azimuth wavenumber on the range direction is removed by an azimuth interpolation. In the conventional PFA processing, a near-uniform *K*_x1_(*m*_1_, 1) is directly used as an azimuth referential interpolation array [1]. However, for the stitched data, serious non-uniformity appears in *K*_x1_(*m*_1_, 1), and, therefore, the azimuth referential interpolation array should be redesigned. By assuming that *K*_xmax_ is the maximum value of *K*_x1_(*m*_1_, 1) and *K*_xmin_ is the minimum value of *K*_x1_(*m*_1_, 1) as shown in Figure 6a, the azimuth referential interpolation array *K*_xref_ yields

(15) $$K_{\text{xref}}\left(m_{1}\right)=K_{\text{xmin}}+\frac{K_{\text{xmax}}-K_{\text{xmin}}}{N_{\text{a1}}}m_{1}$$

where the azimuth number *N*_a1_ is kept unchanged for the azimuth interpolation. The azimuth interpolation converts the signal from the keystone format to a rectangular format (in yellow) as shown in Figure 6b, resulting in a signal format as

(16) $$s_{\text{if}3}\left(m_{1},i_{1}\right)=\exp\left\{-j\left[K_{\text{xref}}\left(m_{1}\right)x_{p}+K_{\text{yref}}\left(i_{1}\right)y_{p}\right]\right\}$$

Inspecting Equation (16), it can be found that the two dimensional signal coupling between the range and the azimuth has been completely removed. Thus, two cascade one-dimensional FFTs can be directly applied to complete the azimuth super-resolution processing, resulting in an enhanced image with a higher azimuth resolution than that acquired by the original stripmap mode as

(17) $$\begin{array}{l} s_{\text{image}}\left({m^{\prime }}_{1},{i^{\prime }}_{1}\right)=\text{sinc}\left[\pi \left({m^{\prime }}_{1}-\frac{K_{\text{xmax}}-K_{\text{xmin}}}{2\pi }x_{p}\right)\right]\text{sinc}\left[\pi \left({i^{\prime }}_{1}-\frac{K_{\text{ymax}}-K_{\text{ymin}}}{2\pi }y_{p}\right)\right]\\ \left({m^{\prime }}_{1}=1,2,...,N_{\text{a}1},{i^{\prime }}_{1}=1,2,...,N_{\text{r}1}\right) \end{array}$$

**Figure 7:**
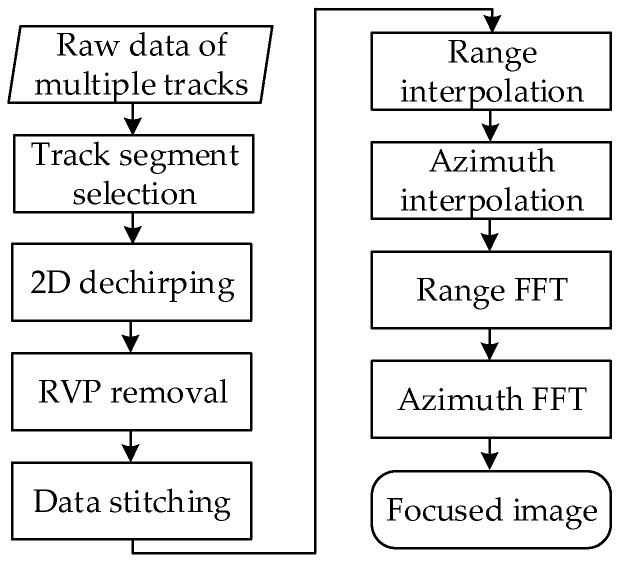
Flowchart of the azimuth super-resolution method.

The flowchart of the azimuth super-resolution method is displayed in Figure 7.

## 4. Restrictions

**Figure 8:**
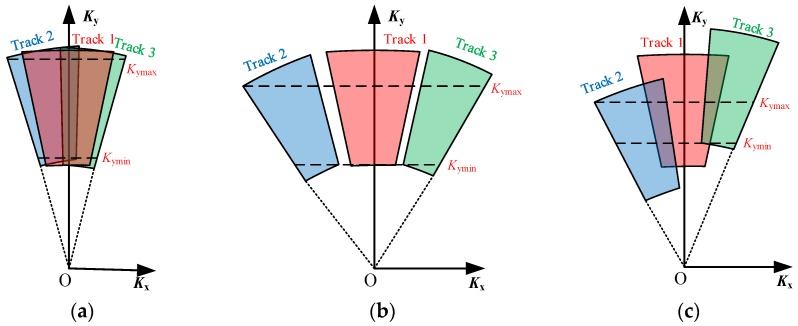
Wavenumber spectrums with (**a**) too much overlapping; (**b**) gaps; and (**c**) too narrow range wavenumber bandwidth.

The effectiveness of the proposed method is sensitive to the geometry of the data acquirement, especially the squint angles and look angles of selected tracks. Firstly, the effectiveness of the proposed azimuth super-resolution method in expanding the azimuth wavenumber bandwidth relates closely to the differential squint angle Δ*φ* between two adjacent tracks. If Δ*φ* is too small, the overlapped area between adjacent wavenumber spectrums will account for a large ratio as shown in Figure 8a, resulting in a shrunken azimuth wavenumber bandwidth and a worse azimuth resolution; if Δ*φ* is too large, gaps are likely to appear between adjacent wavenumber spectrums as shown in Figure 8b, resulting in a deteriorated azimuth focusing quality with expanded mainlobe and increased sidelobes.

A reasonable criteria of the squint angle selection can be set as

(18) $$\left(1-\varepsilon_{1}\right)\theta_{-}{}_{3\text{dBg}}\left(k+1\right)\leq \Delta \varphi \left(k\right)\leq \left(1-\varepsilon_{2}\right)\theta_{-}{}_{3\text{dBg}}\left(k+1\right),\;k=1,2,...,K-1$$

where *θ*_−3dBg_ denotes the beam width projection on the ground as shown in Figure 5a, *K* denotes the number of tracks employed in the proposed method, and *ε*_1_ and *ε*_2_ denote the maximum and minimum wavenumber spectrum overlapping ratios between adjacent tracks. In practical applications, *ε*_1_ can be set as 0.7 and *ε*_2_ can be set as 0.1. Note that the squint angle is comparable to the beam width, and these squint angles should differ slightly from each other. For example, considering an X-band airborne SAR with a 2 m azimuth antenna length and a 45 degree incident angle, a reasonable differential squint angle is 0.7 degrees.

Secondly, the look angle *β*, affected simultaneously by the slant ranges and the track altitudes, determines the distribution of the range wavenumber *K*_y_ as shown in Equation (10). If *β* of selected tracks differ too much from each other, the range wavenumber bandwidth between *K*_ymin_ and *K*_ymax_ applied for the range interpolation will be seriously compressed as shown in Figure 8c, resulting in an undesirably decreased range wavenumber bandwidth and a worse range resolution.

Some other requirements should also be considered. For instance, the parameters in the dechirping filter in Equation (3) should be generated with enough accuracy to completely remove the LFM characteristics of the signal; the sensor position should be measured with enough accuracy to guarantee that the echo can be resorted and stitched correctly; the electromagnetic scattering features of the target region should remain stable to ensure that the echoes acquired by different tracks are well correlated. In summary, the tracks used for the azimuth super-resolution processing should be carefully selected to ensure the effectiveness of the proposed method.

## 5. Simulations

**Figure 9:**
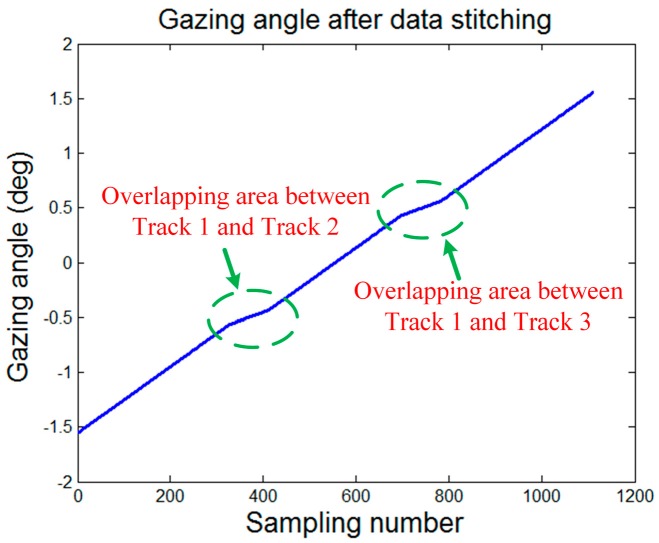
Gazing angle sequence of the stitched data.

The effectiveness of the presented azimuth super-resolution method is evaluated by both the single- and multi-target simulations. The simulations are conducted by hybrid C and Matlab programming. The performances of the focused images using and not using the proposed azimuth super-resolution method are compared. In the experiments, the data of three tracks, acquired by a pulsed X-band airborne SAR in the broadside stripmap mode, are simulated for the data resorting and stitching manipulation. The wavenumber spectrums of the selected data segments are assumed to have a 14% overlapping to generate a continuous stitched wavenumber spectrum. An increasing gazing angle sequence of the stitched data is displayed in Figure 9. The slope where the wavenumber spectrum overlapping occurs tends to be more flattened because the spatial sampling density is equivalently increased due to the spectrum overlapping. The main simulation parameters are listed in Table 1.

**Figure 10:**
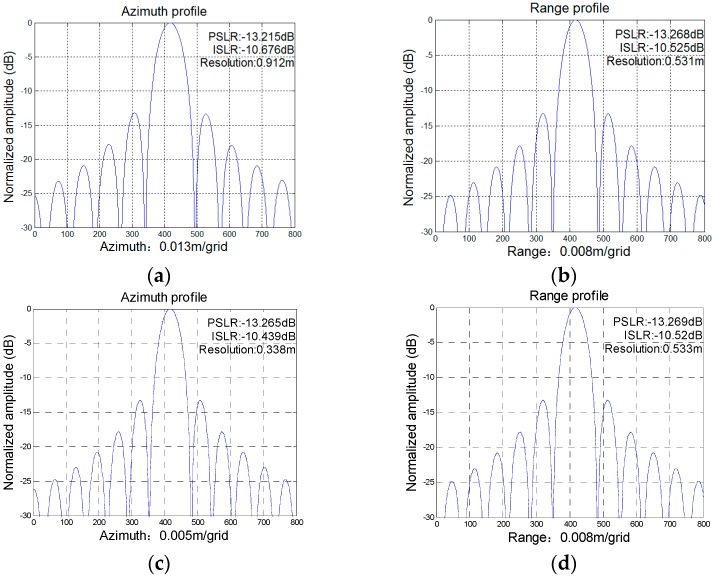
Profiles of the focused single target. (**a**) and (**b**) denote the azimuth and range profiles of the original stripmap data, respectively; (**c**) and (**d**) denotes the azimuth and range profiles of the stitched data, respectively.

In the single-target simulation, the target is assumed to be located at the scene center. Both the range and the azimuth profiles are presented with the performances of the peak side lobe ratios (PSLR), integrated side lobe ratios (ISLR) and measured resolutions. While the measured azimuth resolution of the original stripmap image is 0.912 m in Figure 10a, the azimuth resolution achieved by the azimuth super-resolution method is increased by 2.7 times, to 0.338 m, in Figure 10b. The range resolution of the stitched data (0.534 m) is slightly worse than that of the original stripmap image (0.531 m), which corresponds well with the above-mentioned slight range wavenumber compression in Section 3.

**Figure 11:**
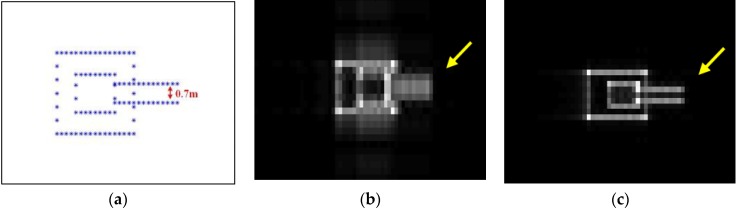
Multi-target simulations of the (**a**) scatterer distribution; (**b**) original single-track image; and (**c**) image processed by the azimuth super-resolution method.

In the multi-target simulation, a double-cannon tank with a size of 8 m × 3 m × 2.5 m is firstly simulated with a 0.5 m interval between adjacent scatterers as shown in Figure 11a. The original single-track focused image is shown in Figure 11b. The image processed by the azimuth super-resolution manipulation is displayed in Figure 11c. It can be noted that, while the two cannons with a space of 0.7 m can hardly be distinguished in the original stripmap image, they can be clearly told apart in the image generated by the azimuth super-resolution method.

**Figure 12:**
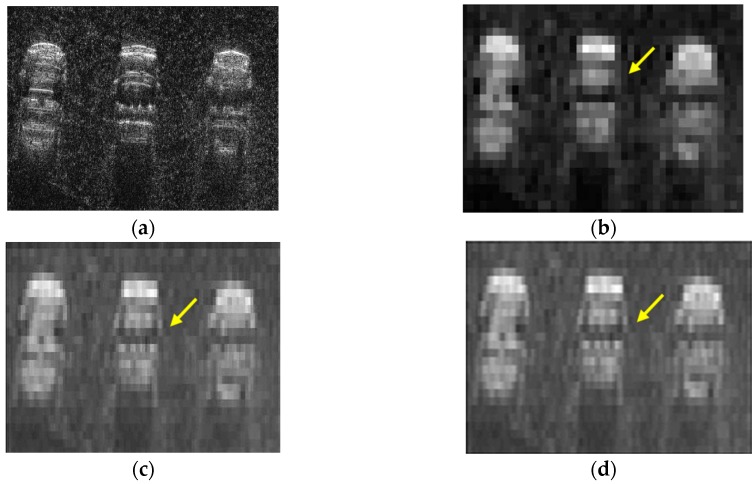
Multi-target simulations of the (**a**) referential airborne synthetic aperture radar image with 0.02 m resolution; (**b**) emulated single-track image; (**c**) emulated image enhanced by the azimuth super-resolution method; and (**d**) spotlight, emulated spotlight image.

Another multi-target simulation is further conducted based on the real airborne SAR images of three vehicles with about 0.02 m resolution as shown in Figure 12a [24]. Figure 12b provides an emulated SAR image focused by a range migration algorithm. The echo of the image in Figure 12b is simulated based on Figure 12a but with parameters in Table 1. The parameters used for this simulation, especially the antenna beam width (deciding the azimuth resolution) and the LFM signal bandwidth (deciding the range resolution), contribute to 0.912 m and 0.531 m resolutions along the azimuth and range directions, respectively, as shown in Figure 12a. As both of the azimuth and range resolutions are much lower than 0.02 m, the resolution performance of Figure 12b is worse than that of Figure 12a. Thus, it is reasonable for this simulation to employ the high-resolution image as an input to simulate the echo and then focus the echo of a single track to generate a low-resolution image in Figure 12b. Figure 12c presents the image enhanced by the azimuth super-resolution method. Comparing with Figure 12b, richer details in azimuth, especially the vehicle gate areas as shown by the yellow arrows, can be noticed. To further illustrate the effectiveness of the proposed method, based on Figure 12a, a spotlight-mode continuous-track echo is simulated, with the same gazing angles as the stitched data and the same parameters in Table 1, and focused by a range migration algorithm, resulting in a focused image as shown in Figure 1two dimensional. Comparing with the images in Figure 12c,d, it is found that the proposed azimuth super-resolution method contributes to a spotlight-mode-level azimuth resolution much better than that achieved by the single-track stripmap mode in Figure 12b. In summary, both the single- and multi-target simulations demonstrate the effectiveness of the presented approach.

## 6. Conclusions

In this paper, an azimuth super-resolution method has been proposed to improve the azimuth resolution of the airborne stripmap SAR image. The main contributions of the presented approach are listed below:

(1) A unique mapping between the time and the frequency domain was realized by a two-dimensional dechirping processing, which lays the foundation for the subsequent data stitching of multiple discontinuous tracks;
(2) A data selection strategy and a data resorting and stitching method in the wavenumber domain were discussed to show how the signal wavenumber spectrum can be expanded in azimuth to achieve a better azimuth resolution;
(3) A two-dimensional data correction method was presented to correct the signal coupling between the azimuth and the range directions.

Future research will focus on the applications of the presented azimuth super-resolution method on real airborne stripmap SAR data.

## Figures and Tables

**Table 1:** Main simulation parameters.

Parameters	Values			
Carrier frequency	10 GHz			
Bandwidth	332 MHz			
Sampling rate	398 MHz			
Pulse repetition frequency	472.5 Hz			
Central slant range	Track 1	Track 2		Track 3
	10.52 km	10.63 km		10.73 km
Central Look angle	Track 1	Track 2		Track 3
	48.3 deg	48.4 deg		48.2 deg
Sensor squint angle	Track 1	Track 2		Track 3
	0.75 deg	0 deg		−0.74 deg
Theoretical ground resolution of original stripmap mode	Range		Azimuth	
	0.53 m		0.90 m	

## Abbreviations

The following abbreviations are used in this manuscript:

SAR	Synthetic Aperture Radar
ROI	Region of Interest
InSAR	Interferometric SAR
RVP	Residual Video Phase
FFT	Fast Fourier Transform
LFM	Linear Frequency-Modulated
TFD	Time-Frequency Domain
PFA	Polar Format Algorithm
